# Palliative care for cancer patients in Sudan: an overview

**DOI:** 10.3332/ecancer.2014.491

**Published:** 2014-12-11

**Authors:** Nahla Gafer, Ahmed Elhaj

**Affiliations:** 1 Clinical Oncologist, Head, Palliative Care Unit, Radiation and Isotope Centre, Khartoum 11111, Sudan; 2 Associate Professor of Oncology, Department of Oncology, National Cancer Institute (NCI-UG), University of Gezira, Hospital Street, PO Box 20, Wad Medani 11111, Sudan

**Keywords:** Sudan, cancer, palliative care, palliative care unit, services, training

## Abstract

Sudan is facing an increasing number of cancer patients every year, and cancer is now among the top ten killer diseases in the country. The majority of cancer patients are diagnosed with an advanced type of cancer where curative treatment has little, if any, effect. The need for palliative care (PC) is urgent. In spite of this, there is no established programme for comprehensive cancer control in the country. In this article we review the state of PC services available for cancer patients.

A PC service started in 2010 as an outpatient service at the main oncology centre in Sudan. With the help of international bodies, several training activities in PC were held. Currently the service includes an outpatient clinic, a nine-bed ward, and a limited home-care service. PC has started to reach two other hospitals in the country.

Unfortunately, the need is still great; the services provided are not fully supported by the hospital administration. And even now, thousands of patients outside the cities of Khartoum and Medani have no access to oral morphine.

## Introduction

The fight against cancer is complex and the increasing burden of the disease makes it one of the most serious health threats to the population in low and middle income countries. Sudan is one of these countries and has been facing an increasing number of cancer patients during recent decades.

Sudan was the largest country in Africa until July 2011 when the South Sudan people voted and separated into an independent country. Sudan now is the third largest country in Africa (after Algeria and the Democratic Republic of the Congo). It is situated in northern Africa at a crossroads between the Horn of Africa and the Middle East. Its 853 km long coastline in the east borders the Red Sea and it has land borders with Egypt, Eritrea, Ethiopia, South Sudan, the Central African Republic, Chad, and Libya.The total population was estimated in 2012 to be 37,195,000, and the total expenditure on health as a percentage of gross domestic product (GDP) was estimated to be 7.3% [1, 2].

The epidemiological profile of Sudan is largely dominated by communicable diseases such as malaria and tuberculosis. In addition to the burden of communicable diseases, Sudan is also experiencing a rapidly increasing burden of non-communicable diseases (NCDs). Of these, diabetes mellitus, cardiovascular diseases, and cancer have been among the top ten causes of hospital admissions and deaths in Sudan since 1998, according to the Federal Ministry of Health. The probability of dying, between ages 30 and 70 years from the four main NCDs which include cancers, diabetes, cardiovascular, and chronic respiratory disease, is 17%. NCDs are estimated to account for 34% of total deaths with cancer causing 5% of the mortality [3].

In spite of the fact that cancer has become among the top ten killer diseases in Sudan, there is no established programme for comprehensive cancer control. The majority of cancer patients are diagnosed with an advanced type of cancer where curative treatment has little if any effect. The need for palliative care (PC) is urgent.

In this article we review the current state of services and progress in education of PC for cancer patients in Sudan.

## Past and present state of palliative care in Sudan

A British PC nurse living in Sudan volunteered in June 2009 to conduct a series of lectures on PC for nurses working at the Radiation and Isotope Centre, Khartoum (RICK) and Soba University Hospital (SUH). In addition, a clinical oncologist and a nurse from the SUH attended a comprehensive five weeks’ initiators course in PC at Hospice Africa Uganda (HAU) in October 2009. Following an advocacy visit from Dr Anne Merriman and Dr Jack Jagwe, an outpatient clinic at RICK started in February 2010, the site of the first PC services in Sudan. Then, a series of PC courses were conducted at RICK and SUH by trainers from outside Sudan. In January 2011 the PC ward was opened with special funding from the African Palliative Care Association.

## Services

Services at RICK include an outpatient clinic open five days a week, accepting patients from other oncology units at RICK (about 70% of referrals) or from outside the hospital (30%) (Table 1). All referred patients are clerked and then receive management on the first day of their arrive. Most of the patients are seen in the outpatient clinic with an average frequency of six visits per patient. Patients are seen about once every two weeks.

The ward with nine beds acts as a demonstration site for holistic nursing care, continuity of care, networking, and effective communication with the patient, and patient’s family members. Patients are usually admitted for symptom control or end of life care. The majority of admissions are for a duration of less than five days.

A limited home-care service was started in May 2011. On request of the families, the PC staffs volunteered to conduct home-care once a week usually on Saturdays (the reason being less traffic on this day). The patients’ families provide the vehicle because currently there are no dedicated cars provided by the hospital.

## Major changes at the hospital practice (RICK)

In the palliative care wards, nurses and doctors train the caregivers on how to look after and perform dressing of fungating wounds, give medications, get physiotherapy, receive healthy nutritional advice, and general care for the bed-ridden patients; resulting (ironically) in that the PC wards have the least mortality compared to the other oncology wards.

The palliative care unit (PCU), since its inception, introduced major changes in clinical practice, and the examples include:

- Symptom burden in patients with advanced cancer (emotional and spiritual distress) have been addressed.
- Proper use of morphine tablets (prior to opening the PCU, immediate release tablets were mistakenly used twice a day).
- The introduction of liquid oral morphine: initially the morphine vials for injection were reconstituted by adding drinking water and for the last year ready-made liquid morphine was imported. Unfortunately, oral morphine is still not available outside the three centres in Sudan, and also not outside official work hours.
- Addressing social issues: many wives are abandoned because husbands believe cancer is incurable or even contagious.
- Addressing psychological issues: for example, many female patients had a fear of touching their chest after mastectomy or washing the area even a long time after surgery.
- Sometimes the carer would not touch the patient; this is something that would change after seeing doctors and nurses sitting by the patient and touching them.
- Training and helping caregivers who are facing a lot of problems looking after loved ones.
- Continuity of cancer care (including, for example, contacting patients by phone—a new practice in the hospital).
- Referral and inter-displinary consultations: having specialists from other hospitals come and see patients at the PCU, e.g., maxillofacial surgeons, neurosurgeons.
- Use of metronidazole crushed tablets for fungating wounds (a practice exported from HAU). Also the use of these tablets, i.e., when they are to be inserted twice daily per vagina for patients with cervical cancer, reducing greatly the general bad odour in the wards.
- Training of the patients and their caregivers on how to perform wound dressing simply at home.
- Good communication skills, creating longstanding, strong, and good relationship between patients and the PC team members which help in clarifying patients’ understanding of their prognosis and empowering them to make treatment and life-changing decisions.
- Telling the truth about the status of the disease gives patients a chance to disclose very important issues in their lives and prevents a conspiracy of silence during those important days of life; this also prevents trends of futile treatments including continuous searching for costly treatment inside the country or abroad.
- Providing for privacy and time at the outpatient clinic. At oncology clinics, patients are seen together, usually two patients at the same table with an average follow-up visit of 5–10 minutes. At the PC clinic, patients are seen separately, with an average time of 15–20 minutes for a follow-up visit and one hour for clerking.

All these interventions led to better outcomes and improved quality of life to a degree that patients started asking when they can be transferred to the PCU.

## Services outside RICK

- At SUH since 2009, there is a team consisting of a doctor and a nurse. They have recieved some support from the hospital administration. They offer consultation services to inpatients at the Surgery and Medicine Departments. Oral morphine has been made available to inpatients.
- National Cancer Institute, Medani (NCI). Efforts are in place to start in a few months’ time. Several staffs have been trained in PC, and an enuthasiastic nurse has even started a wound clinic.

### Statistics about the PCU at RICK (from a total of 1,249 patients) [4]

1. **Distribution of patients according to residence:** 12% from Khartoum State; 1% Foreigners; 87% from outside Khartoum state, mainly Darfur and Kordofan states. 80% of patients are of low socio-ecomonic status.
2. **Sex distribution:** 58% females; 42% males.
3. **Age distribution:** Average age: 51 years. 6% are children (less than 16 years of age).
4. **Distribution according to diagnosis:**
  **Table:** 

	Site	%
1	Gastrointestinal tract (HCC 10%; Stomach 6%)	27
2	Gynaecological (Cervix 7%)	12
3	Breast	10
4	Head and neck	7
5	Urological	7
6	Metastases of unknown origin	6
7	Soft-tissue sarcoma	6
5. **Major Symptoms:** The average period of follow-up was eight weeks, which might reflect families’ decision to return back to their home-land.
  Patients usually die within the first eight months from referral to PC. Breast cancer patients and paediatric patients generally survive longer periods (years).
  **Table:** 

	Symptom	%
1	Pain	91
2	Nausea	40
3	Vomiting	37
4	Shortness of breath	25
5	Neurological symptoms	25
6	Constipation	24
7	Offensive wounds (All carers were trained how to do Flagyl dressing)	24
8	Diarrhoea	15
9	Anxiety	13
10	Psychological problems	12
6. **Major treatments recieved:**
  **Table:** 

	Treatment	%
1	Morphine	71
2	Tramadol	36
3	Bisacodyl	36
4	Metcholopramide	32
5	Dexamethasone	30
6	Loperamide	14
7	Counselling (In fact, 56% of patients do not know the prognosis on presentation to the unit; 44% do not even know the diagnosis)	53
  Of all patients, 54% recieved some form of chemotherapy (all provided at RICK for free); 38% recieved radiotherapy.
7. **Presence of metastasis:** 35% of patients had liver metastasis; 27% had lung metastasis; 15% had bone metastasis; and 6% had brain metastasis.
8. **Follow-up and feedback:** More than 20% of families were contacted by the team after the patients’ death; and more than 15% of patients or families expressed spontaneous positive feedbacks.

## Palliative care training and education

**Figure 1.**
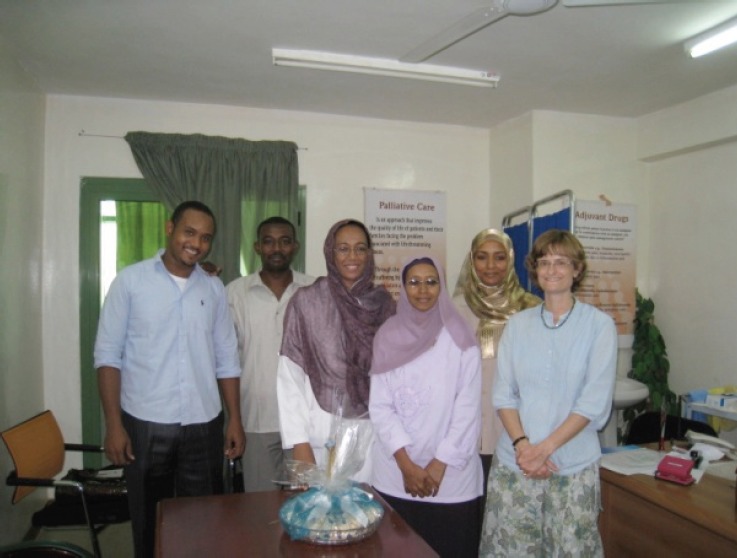
The first PCU team members—RICK, 2010.

Four members attended PC courses at HAU, Kampala, through funding from the International Association of Hospice and Palliative Care; and also attended another course at Kuwait, Palliative Care Centre, which was funded through the World Health Organisation (WHO) (Figure 1).

There are now 30 health professionals passed by the PCU for different periods of training (usually more than five months): they include doctors, nurses, nutritionist, psychologists, and social workers (Table 3).

## Conclusion

In spite of the urgent need for PC in Sudan, the available services exist only in three institutes. There are limited PC services at RICK, SUH, and NCI. International PC associations had a strong hand in backing these services. The lack of oral opioids elsewhere limits pain management. Palliative care in Sudan is a fairly new concept but has proved worthwhile in alleviating patient’s symptoms and helping families of patients with cancer. A lot of effort is requested from stakeholders to expand and cover all those who are in need.

## Abbreviations:

HAU: Hospice Africa Uganda, Kampala
ICPCN: International Children’s Palliative Care Network
IAHPC: International Association of Hospice and Palliative Care
MPCU: Makerere Palliative Care Unit, Kampala
NCI: National Cancer Institute, Medani—the second oncology centre in Sudan, situated at Gezira state and affiliated to Gezira University
PC: Palliative Care
PCU: Palliative Care Unit
RICK: Radiation and Isotope Centre, Khartoum—the main oncology centre in Sudan, situated in the centre of the capital
SUH: Soba University Hospital, secondary referral hospital south of Khartoum with more than 700 beds.

## Authors’ statement of contributions

Both authors contributed equally to the manuscript.

## Figures and Tables

**Table 1.** Status of services delivered up to July 2014.

	Outpatient clinic	Inpatient admissions	Home-care
No. of patients	1,249	680	344
Start of service	Feb 2010	Jan 2011	May 2011
Frequency	Daily office hours	Daily into nine beds	Once a week

**Table 3.** The following training activities took place inside Sudan, in addition to on-the-job training.

N	Event, period	No of Candidates	Support
1	Introduction to PC for health professionals, a four week course March 2010	24 doctors and nurses	HAU
2	Introduction to PC for volunteers, a four days course, March 2010	32: from NGOs, pharmacists, students of medicine	HAU
3	Palliative care in practice, a three-days’ workshop, Dec 2010	36 mainly staffs from Soba University Hospital	
4	Advanced Palliative Care, a one week workshop—in Khartoum	17 team members working at RICK–PCU	HAU
5	Introduction to PC, a one week course—at Medani	25 health professionals working at NCI	HAU
6	First paediatric PC workshop, one week, May 2013	37 health professionals from five different hospitals	ICPCN
7	The PC approach in cancer management, two week workshop, July 2014, in Khartoum	25 health professionals: oncologists, surgeon, medical officers, nurses, pharmacists and psychologist	MPCU
